# Maximising contributions of midwives in Africa towards achieving MNH targets: Lessons learned

**DOI:** 10.4102/phcfm.v17i1.4851

**Published:** 2025-04-16

**Authors:** Doreen K. Kaura, Jemima A. Dennis-Antwi, Frances D. Ganges, Sarah N. Ngoma

**Affiliations:** 1 Department of Nursing and Midwifery, Faculty of Medicine and Health Sciences, Stellenbosch University, Cape Town, South Africa; 2 Centre for Health Development and Research (CEHDAR), Accra, Ghana; 3 Jhpiego, Johns Hopkins University Baltimore, Maryland, United States; 4 Midwives Association of Zambia, Lusaka, Zambia

**Keywords:** primary care, maternal, neonatal, midwifery, human resource

## Abstract

African midwives are pivotal in enhancing continuity and care coordination throughout healthcare systems. They are a critical human resource in mitigating near misses, morbidities and mortality in Maternal and Neonatal Health (MNH). Thus, achieving the sustainable development goals (SDGs) necessitates robust midwifery policies and system strengthening across Africa. Given the critical role of midwives, this report reflects on the need for a coordinated regional approach to unify midwifery across the continent as a strategy towards impactful SDG achievements. The reflections are based on insights from a Ghana meeting aimed at unifying African midwives. Globalisation and Africanisation are both crucial for developing a harmonised sexual, reproductive, maternal, newborn and adolescent health system that improves the quality of life for African women and their families. Despite significant healthcare improvements, Africa faces challenges such as a shortage of skilled birth attendants, leading to high maternal and neonatal mortality rates. Achieving SDGs requires local solutions and fully integrating midwives into health systems. It also requires that midwives are purposefully and regularly engaged in global, regional and local policy discussions and decisions. To support this goal, the authors present an approach to ensure midwives in Africa are not only represented in these forums but also actively engaged in shaping, advocating for, and advancing relevant actions. We therefore recommend establishing a regional midwifery body to lead and coordinate these efforts.

## Introduction

We argue that globalisation is imperative, but so is the need for Africanisation – African perspectives by Africans. Africanisation is required for efforts towards a harmonised Sexual Reproductive, Maternal Neonatal and Adolescent Health (SRMNH) system that ensures an improved quality of life for all African women and their families through improved access. This brings us to the question: Is Maternal and Neonatal Health (MNH) in Africa an absolute challenge, or is it a relative confrontation of perceptions created by a historical stereotyping of Africa as an overwhelmed continent? There is no doubt that MNH remains a significant public health challenge in Africa. However, the shortage of skilled birth attendants^1,2,3^ drives high maternal and neonatal near-miss cases and mortality rates, which continue to plague many countries despite significant improvements in healthcare delivery.^3,4^

Sustainable development goals (SDGs) to improve MNH in Africa require well-thought-out actions with the application of home-grown solutions and the adaptation, adoption and translation of global solutions in a way that effectively addresses the African challenges. This calls for a unified African voice in search of local solutions especially by skilled health workforce who are near and integrally involved in the care of mothers and their families. The midwife is a prime representative addressing MNH challenges in Africa who requires strong localised advocacy efforts,^4,5,6^ particularly in shaping Human Resources for Health (HRH), policies and integrating midwives into health systems more effectively.

## Unifying midwifery in Africa to achieve sustainable development goals

With about 5 years remaining until 2030, African governments are striving to move closer to their health and development goals. Several countries at the 2024 World Health Assembly (WHA) passed a critical resolution committing to specific actions that are well aligned with those of the WHO Global Strategic Directions for Nursing and Midwifery 2021–2025^7^ and the State of the World’s Midwifery (2021).^8^ Two of these actions highlight the pivotal role of midwives and midwifery care:

- Provide required human resources, including through *midwifery models of care*.
- Invest in the education, employment, regulation and retention of the workforce including midwives and nurses.

Undeniably, educated and supported midwives are essential to national and global efforts in achieving health goals and improving outcomes for mothers and their newborns. But how can we ensure that midwives are central to relevant policy deliberations, discussions and decisions? And that African midwives are represented at global and national forums.

It is against this backdrop that midwifery leaders from Africa and the diaspora converged in Ghana in May 2024 to deliberate and make recommendations for the creation of a formidable continental midwifery organisation. Such an organisation would serve as the voice for African midwives and the midwifery profession. The aim is to mobilise and unite African midwives to translate and culturally adapt the global health agenda to align with evidence-based national interventions and ensure that midwives optimally contribute to achieving the SDGs and beyond.

## Midwives and maternal and neonatal health

Sustainable Development Goal Target 3.1 calls for all member countries to reduce maternal mortality to less than 70/100 000 live births by 2030.^9^ However, in 2020, the WHO reported that pregnancy and childbirth-related conditions resulted in the loss of lives for about 800 women daily across 185 countries globally.^9^ Fortunately, the evidence is clear: the availability of competent midwives who are (1) educated based on global standards; (2) regulated by a strong regulatory body; (3) licensed to practice within a multidisciplinary team; and (4) supported with an enabling environment could avert over 80% of these deaths through their timely interventions.^9,10^ This alone makes a strong case for stakeholders to invest in midwives through relevant policy and practice reforms to reposition, strengthen and support robust midwifery education, regulation and practice.

As the voice of the profession, midwifery associations advocate enhancing their cadre to promote high-quality SRMNH services through autonomous, responsible and accountable midwives working within their full scope of practice.^11^ While organisations such as the International Confederation of Midwives (ICM) aim to support this globally, we maintain that a unified and harmonised approach is needed specifically for Africa – one that both reaches and represents every country and every midwife on the continent.

Midwives deliver a full range of quality interventions across the continuum of care. When their capabilities are effectively harnessed, they improve over 50 health outcomes. These include reduced mortality and harm to women and newborns, better psychosocial well-being, and enhanced public health and healthcare services.^12^ Moreover, reflecting on Africa being prone to civil and humanitarian crises, the increasing importance of midwifery practice in humanitarian and climate-ravaged communities is an emerging and vital role. As a matter of urgency, MNH stakeholders must refocuss on how best to ensure that midwives are educated according to global standards and supported to be in the right places, at the right time, with the right resources, and competently practising as fully responsible and accountable professionals.

## Unifying midwifery in Africa: The need for a regional entity

According to the ICM, the three key pillars of a profession are education, regulation and professional association. These must be supported by strong policies, leadership, sound governance and a formidable workforce management system within an atmosphere of respect for the voices of midwives for mothers, their newborns and the profession of midwifery by midwives. While the first two are the purview of the national government, the association is typically the voice of the profession, playing a central role in advocacy and overall efforts to promote and improve the profession. Associations champion their professions by providing resources, information and opportunities for professional development – often working in tandem with education, regulation, clinical services and research colleagues – all with the aim of helping its members to provide the best quality care. Midwives’ associations around the world include local, national, regional and sub-regional organisations. The Ghana Registered Midwives Association and the Uganda Private Midwives Association are among the oldest such organisations in Africa.

In May 2024, a summit was convened by African midwives and their stakeholders, with the theme ‘Unifying Midwifery in Africa: Reimagine, Reunite, Rise’. The purpose was to facilitate strategic deliberations and discussions on the state of midwifery in Africa and explore the need for a unified entity. Summit participants proposed that such an organisation could be the leading professional body to mobilise, unite and represent African midwives. In addition to advocacy, this entity would also serve to provide educational and other resources and support efforts to enhance midwifery around the region. Participants further envisioned identifying midwives across the continent to fill the roles of consultants, researchers, and educators for projects and organisations focussed on MNH. It was agreed that this proposed body would not eliminate the need for existing organisations, but instead align with and support common objectives.

To realise these aims, participants developed a communique, spelling out actions and next steps to establish the proposed entity. This ‘roadmap’ is expected to be completed by late 2024 and implemented over the next 2–3 years.

## Conclusion

A unified African front, in collaboration with country midwives, health systems, regional bodies, and partners, can drive investments in midwifery. These investments will help achieve the SDGs and other health targets while improving maternal and neonatal health for future generations.
